# A Cohort Study Analysing the Impact of the COVID-19 Pandemic on Colorectal Cancer Presentations in a Medium-Large Canadian Community Hospital

**DOI:** 10.1177/10732748251356926

**Published:** 2025-07-02

**Authors:** Erik Cassar, Caroline Hamm, Rong Luo, Ilinca Georgescu, Akmal Ghafoor, Sumin Rhee, Fawad Ahmed

**Affiliations:** 1 Schulich School of Medicine, 8637 University of Windsor, Windsor, ON, Canada; 2 8636 Windsor Regional Hospital, Windsor, ON, Canada

**Keywords:** colorectal cancer, covid-19, colorectal cancer screening, pandemic, colon cancer

## Abstract

**Introduction:**

Colorectal Cancer (CrC) is a common cause of cancer-related death worldwide, but screening programs are highly effective at diagnosing early-stage disease, allowing effective treatment. During COVID-19, a decrease in screening participation was hypothesized due to limited access, leading to an increase in symptomatic presentations and stage at diagnosis.

**Methods:**

All patients who met inclusion criteria were divided into two cohorts based on time of diagnosis (*n* = 373). The pre-COVID era was designated as December 2018 to February of 2020, with the COVID era running from then until March 2021. All patients were from the Windsor Regional Hospital Cancer Centre, located in Windsor, Canada.

**Results:**

Across time periods, 218 patients were diagnosed prior to, and only 144 during COVID. The number of Fecal Immunochemical Test (FIT) positive patients remained stable, while the number of procedural diagnoses decreased from 34.1% to 10.7%, with only 21.2% of patients overall being diagnosed with screening. When combining time periods, females presented symptomatically (85.0%) more often than males (74.4%). Patients with a positive family history were more likely to be diagnosed via procedural screening (42.9%) than those without (20.4%).

**Conclusion:**

There was no change to the proportion of symptomatic presentations across time groups, in contrast to our predicted outcome. There was a decrease in procedural screening during the COVID timeframe, with FIT testing rates remaining stable, likely representing patients being transferred to available methods. Female patients and patients with a family history demonstrated a particular need for increased screening participation based on our findings.

## Introductions

Colorectal cancer (CrC) is the third most commonly diagnosed cancer in Ontario and Canada, and fourth most common in the world, making it the second most common cause of cancer related death in Canada, and the third deadliest cancer worldwide.^1-3^ In order to combat the impact of colorectal cancer related morbidity and mortality, screening programs have been broadly adopted by many countries around the world.^3^ These measures have demonstrated to be very effective at reducing the stage of disease at diagnosis, and allowing for earlier and more effective treatment for patients.^4,5^ According to Cancer Care Ontario, nine out of ten deaths from colorectal cancer can be avoided with effective screening.^6^ Between the years of 1987 and 2010 in the United States alone, screening rates increased from 34.8% to 66.1%, leading to a decreased incidence of late-stage disease presentation from 118 to 74 cases per 100 000.^4^ In Canada, screening rates have increased from 42% in 2010 to 54% in 2017, with a target of 60%.^7^ Patients in Ontario are categorized into average risk and high risk, based on a number of factors. Screening is recommended for all average risk Ontarians between the ages of 50 and 74, with a Fecal Immunochemical Test (FIT) test every two years or colonoscopy every 10 years. High risk patients, such as those with an immediate family history, have a more stringent screening protocol.^8^

In 2020, many countries began to implement sweeping public health measures in response to the spread of COVID-19, which affected the delivery of healthcare. During the initial waves of the pandemic, it is generally believed that many patients were fearful to attend primary care appointments and, presumably, participate in colorectal cancer screening due to concern of contracting COVID-19. This study aimed to characterise whether there was a decrease in adherence to, and by extension the effectiveness of, colorectal cancer screening by comparing data from before and during the pandemic. Other studies have analysed similar research questions, with differing results.^9^ One such article described a decrease in FIT testing by 85%, and a decrease in procedure-based screening (such as colonoscopy or flexible sigmoidoscopy), by 90%.^10^ By comparison, most other studies showed a smaller decline in FIT testing, demonstrating a larger gap between FIT testing and procedural screening. One study demonstrated a 62% reduction in colorectal cancer diagnoses, resulting from an 87.8% reduction in screening participation.^11^ The findings of decreased colorectal cancer diagnoses were also supported by a single study performed at a tertiary Canadian oncology centre.^12^ One study performed in Quebec demonstrated a decrease by 67% in FOBT (similar/equivalent to FIT) testing and 58% decrease in procedural screening.^13^ In essence, other similar studies demonstrated a reduction in colorectal cancer screening participation and effectiveness, albeit with differing magnitudes, potentially leading to increased morbidity and mortality related to colorectal cancer as a result of pandemic related changes to screening delivery and patient hesitancy for seeking care. Our prediction is that the number of new diagnoses in the months to years following the COVID-19 pandemic will increase compared to previous, to account for the decreased numbers during this time period, as the true incidence of colorectal cancer likely did not change, only our ability to detect it.

While these other studies demonstrated some consistencies with our results, our study is unique in that it encompasses the entire diagnosed population of a medium sized Canadian city. Additionally, this study aims to contribute to a growing body of evidence demonstrating the impact of pandemics and interruptions in healthcare on cancer screening and the population. Our conclusions may help to influence public policy for future pandemics, as well as the messaging and recommendations from public health on the importance of maintaining primary care and screening programs. Shadowing the primary aim of this study, our data is likely to serve as a demonstration of the current screening rates and effectiveness of the colorectal cancer screening program as well. It may help to guide new initiatives aimed at increasing participation across Ontario and improving the resiliency of screening programs.

## Methods

The target population of the study was based around our local regional cancer centre, in Windsor-Essex County, Ontario, Canada. Upon approval from the institutional research ethics board (approval #21-399), we were able to collect and assess data on all patients who met our inclusion criteria and were referred to the cancer centre for systemic treatment. Their health information was accessed through Cerner and Solcom, the Electronic Medical Record (EMR) systems in use within the Windsor Regional Hospitals. During the timeframe of the study, the switch was made from Solcom to Cerner, requiring access to both databases. A list of all patients who were referred to the Windsor Regional Cancer Centre for colorectal cancer primaries was acquired, and these patients were divided into cohorts based on time of diagnosis. Patients were excluded if the reason for referral was not for colorectal cancer primary, or if it was a recurrence of previous CrC. The timeframe of the study stretched from the first of December of 2018, to the thirty first of March of 2021, with the months between the first of February 2020 and the thirty first of March 2021 being designated as the COVID-19 timeframe. If the patient had undergone initial FIT testing which was positive, and then proceeded to a colonoscopy, the patient was counted as had having a FIT test since this was how the cancer was initially detected. Patients who presented with symptoms and received a colonoscopy to confirm diagnosis were considered to be symptomatic, as were patients diagnosed with imaging. For the purposes of this study, flexible sigmoidoscopy was considered equivalent to colonoscopy and they were grouped as procedural screening. Date of diagnosis was based on the pathology report date when available, otherwise the best estimated date of diagnostic confirmation was used. Depending on the scenario, this may have been the date of colonoscopy confirmation, abdominal Computed Tomography imaging which demonstrated bowel obstruction, or similar. The population of this region increased by 5.82% from 2016 to 2021, the years where census data was collected. Given the very small increase in population on a yearly basis, it was assumed that the population growth itself was not a significant contributor to the incidence of colorectal cancer in pre vs pandemic era comparisons.

The study aimed to measure the rates of symptomatic presentations compared to positive screening with either FIT, colonoscopy or flexible sigmoidoscopy. This was accomplished by analysing patient charts and reading physician notes in order to collect data on method of diagnosis, stage at diagnosis, among many other data points. Screening methods were analysed independently, in order to determine if each were affected differently during the pandemic. Other data were collected including demographic data, stage of primary tumor at pathology diagnosis, location of tumor, family history status, and personal history status of inflammatory bowel disease and colorectal cancer.

Our inclusion criteria excluded all patients whose tumors were successfully resected during colonoscopy screening, as patients with small local tumors and negative margins are not referred to the regional cancer centre for further treatment. Of note, these criteria also excluded those with a positive FIT who were referred for colonoscopy who also had complete resection of the polyp/tumor. However, the principal purpose of the study was to identify how COVID-19 related restriction on health care access impacted the presentation of clinically advanced colorectal cancer cases. Since this study compares a time period prior to COVID with a similar time frame during the COVID era, the confounding variable of missing early-stage disease due to not being referred to the cancer centre should be balanced. Nonetheless, this sample bias was carefully considered in the analysis and conclusions that can be drawn from the data collected in this study.

The reporting of this study conforms to STROBE guidelines.^18^

## Statistical Analyses

Statistical analyses were performed on the data to draw comparisons between the time periods, but also between demographic groups. Continuous variables such as age were expressed as mean ± standard deviation and were compared using an independent-samples t-test. Categorical data were expressed as numbers with percentages and were compared using cross tabulation with chi-squared tests. All *P*-values were from two sided tests, and results were considered statistically significant if *P*-values were less than 0.05. Analyses were conducted using IBM SPSS 20 statistical software, using a combination or logistic, ordinal logistic, and multinominal logistic analyses. Overall, we did not identify any confounding factors requiring statistical adjustments.

## Results

Data on demographics were collected in order to identify any differences in patterns between certain demographic groups. The proportion of men and women were similar across the time periods of comparison, as was the age distribution (Table 1). The number of patients presenting with a positive family history remained similar; however, there was a large drop in the number of participants without family history of colorectal cancer during the COVID era time period (Table 2). Despite this, the proportions are not significantly different.

**Table 1.** Age Demographics of Patients Presenting With Colorectal Cancer Prior to and During the COVID-19 Pandemic

Age	Before COVID	During COVID
30-39	4	2
40-49	15	5
50-59	40	32
60-69	57	41
70-79	63	39
80-89	41	28
90-99	5	2
*P* value	0.82	

Age distribution demographics for entire study cohort, with no statistical difference between time groups (*P* 0.82 using Welch Two sample T Test).

**Table 2.** Sex Demographics and Colorectal Cancer Family History Status of Those Presenting With Colorectal Cancer Prior to and During the COVID-19 Pandemic

Males	Females	Proportion	*P* value
122 Before COVID	103 Before COVID	1.18 to 1	0.14
90 During COVID	59 During COVID	1.52 to 1	
Positive family history of CrC	No family history of CrC	Proportion	*P* Value
44 Before COVID	175 Before COVID	1 to 0.25	0.25
36 During COVID	107 During COVID	1 to 0.34	

Comparison of proportions between time periods of male to female, and positive family history to no family history. The sex demographic comparison does not have any missing participants, and there was no statistical difference between the time groups (*P* 0.14 using a Pearson Chi-Square Test). For family history demographics, total patient number was 362; 12 patients were omitted for missing data with no significant difference between time periods (*P* 0.25 using a Pearson Chi-Square Test) for change in proportion of participants with family history.

While overall ratios of screening vs symptom presentation did not change between the time periods, methods of screening that were performed did change. Rates of colonoscopies dropped significantly, representing a drop from 34.1% of the screening group to only 10.7%. By comparison, FIT testing had no change in number of patients (*P* 0.02), constituting pre COVID percentage of 65.9% to COVID of 89.3%. Proportion of FITs increased, statistically significantly, but not clinically because there was no increase in the actual number of positive FIT tests (27 before COVID and 25 during COVID). The increase in proportion was driven entirely by the decrease in colonoscopies since the participation in FIT testing held steady. In total, few patients requiring systemic treatment were diagnosed with screening, representing only 20.27% and 22.14%, before COVID-19 and during the pandemic respectively (*P* .38 for difference between time periods).

When comparing based on sex, females and males had no difference for screening type based on our data, showing a combined 75.4% preference for FIT testing (*P* .61 for difference between men and women). However, there was a significant difference between sexes on presentation, with females more likely to present with symptoms rather than through screening (85.0% compared with 74.4%; *P* .01).

For patients with a positive family history, there was no difference between preference for procedure based screening or FIT based screening in those who presented with positive tests requiring treatment at the cancer centre; however, the proportion of those using colonoscopies with a positive family history was much higher than those without (42.9% vs 20.4%). When examining the overall screening rates of those with a family history across time periods, there was no change, despite a drop in the use of colonoscopies. This indicates that these patients were likely switched to FIT testing based on lack of availability of the preferred procedural screening methods for this population during the COVID era.

## Discussion

Since their introduction, screening programs have resulted in a significant reduction of mortality and morbidity related to colorectal cancer.^4,5^ In order for these programs to be effective, however, they require robust participation and careful follow-up. The COVID-19 pandemic affected the delivery of healthcare services in ways that are mostly unprecedented in modern medicine. Our study aimed to elucidate if the participation in, and subsequent effectiveness of, colorectal cancer screening programs were affected by the COVID-19 pandemic. Secondarily, we sought out to identify if there were any specific demographic groups that may have been impacted more dramatically.

**Figure 1.**
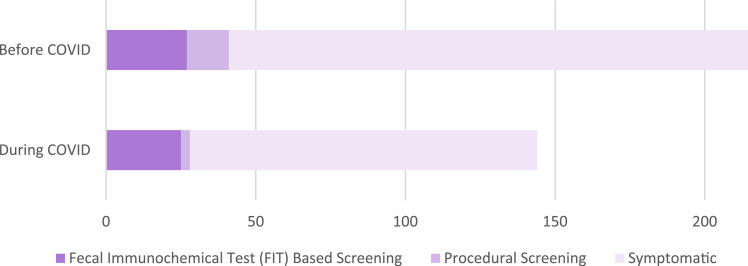
Method of Colorectal Cancer Patient Diagnosis Compared Between the Pre-COVID-19 Timeframe and the COVID-19 Timeframe. Comparison of Patient Presentation Across Time Groups, Demonstrating a Decrease in Procedural Screening Methods. For this Comparison, 11 Patients Were Omitted for Lack of Data. Before COVID, 27 Patients Presented With FIT, 14 With Procedural Screening, and 177 as Symptomatic. During COVID, 25 Patients Presented With FIT, Compared With 3 for Procedural Screening, and 116 Symptomatic Presentations. *P* Value of .024

This study used data from the Windsor Regional Cancer Centre EMR to analyse all patients diagnosed with a primary colorectal cancer within the timeframe of the study. The study timeframe months were divided equally between the pre COVID and COVID eras. Our initial predictions presumed a substantial increase in the proportion of symptomatic presentations during the COVID-19 timeframe. This was predicted due to the idea that there would be a decline in the participation in screening programs during the COVID era, which would have led to a higher representation of symptomatic presentations. However, this was not represented in our data, which demonstrated that 177 people presented symptomatically prior to COVID, with 116 presenting during the COVID era, representing 79.7% and 77.9% of total diagnoses, respectively (*P* .38 showing no difference between time periods) (Figure 1). This finding may be due to the end point of the study not being far enough out from the COVID era to capture the predicted increase in late-stage disease, as well as overall presentations, to account for the decrease in diagnoses within the COVID timeframe. Our data did show a decrease in procedure-based screening methods, such as colonoscopies, in line with other similar studies performed in the United States.^14-16^ Prior to the COVID pandemic, 34.1% of patients were diagnosed using procedure-based screening, compared with 10.7% during the pandemic (Figure 1). In contrast, our data shows that FIT testing did not decline in use or effectiveness during the pandemic (Figure 1). In all, 27 patients were diagnosed with FIT testing prior to COVID, and 25 patients during the pandemic, representing an increase from 65.9% to 89.3% of all screening diagnoses (Figure 1). Despite the similar absolute values, the increase in proportion of FIT testing leading to diagnosis likely represents patients being moved from colonoscopy-based screening to FIT testing based on availability. Our data is in slight contrast to some other similar studies, which demonstrated a reduction in overall screening, including colorectal, breast, and prostate, by 60-80%.^1^ While our study population did demonstrate a decrease in procedural screening, the overall screening did not decrease dramatically, with 20.3% of patients prior to COVID being diagnosed with screening compared with 22.1% during (Figure 1). This may be due to a variety of factors, including differences in access to FIT testing, in screening guidelines across countries, and differences in access to healthcare or cost of obtaining appropriate screening or assessment. In Ontario, reminders are sent to patients for FIT testing independent of their physician, which is not common practice in other countries such as in the US where one study demonstrated a large decrease in FIT testing during the pandemic.^10^ The promising findings from our study population demonstrate the utility of FIT testing particularly in times or environments where procedural screening may be difficult to perform, may it be during a global pandemic or in remote parts of Canada. Despite a drop in colonoscopies, the data suggests that FIT testing is as effective as colonoscopies in the detection of colorectal cancer in our study population due to the maintenance of the screening proportion of diagnoses.

Overall, data collected in this study demonstrated that the participation in colorectal cancer screening programs is not as high as we had initially predicted, with only 21.0% of patients averaged across time periods being diagnosed with screening (Figure 1). It was also noted that the number of advanced symptomatic presentations, such as complete bowel obstructions, were not an insignificant proportion. It is possible that the high representation of un-screened patients in the cohort of our study is due to the inherent limitation of our study in using data exclusively from patient presentations to the cancer centre. Effectiveness of CrC screening at detecting and preventing colorectal cancer can be presumed because those receiving regular screening were detected in pre-cancerous/early-stage disease, and were able to have polyps removed without having to be referred to the cancer centre for systemic treatment consideration. However, as mentioned previously, the principal purpose of the study was to identify how COVID-19 related restrictions on health care access impacted the presentation of clinically advanced colorectal cancer cases.

As was acknowledged widely during the COVID-19 pandemic, there was a reduction in the number of people presenting to healthcare for routine care, as demonstrated by the decrease in overall patient numbers during the COVID timeframe of the study. However, our data does not demonstrate a statistical difference between the number of symptomatic presentations between time groups, likely due to patients with concerning symptoms being compelled to present despite the risks of COVID-19. During the COVID-19 pandemic, the emergency room was consistently open and so patients had the ability to present after developing symptoms. This is represented as 79.7% of patients in the symptomatic group before COVID, compared with 77.9% during the pandemic, demonstrating no statistical difference between the time periods (*P* .38) (Figure 1).

When looking at the entire population of the study, there were some interesting demographic findings. In the subgroup of patients who had information available about family history of colorectal cancer, screening colonoscopies were not the preferred method of screening, at only 42.9% (Table 3). This is in contrast to the current recommendation from Cancer Care Ontario, which states that high risk patients should be screened with colonoscopy or sigmoidoscopy, rather than FIT testing.^6^ However, the proportion of colonoscopies is still much higher in this group than the cohort without a positive family history, indicating that there is still a higher preference for procedural screening in this population as per best guideline recommendations. Demonstrating a similar trend to the overall patient population, the cohort of positive family history also demonstrated a switch from procedural screening to FIT testing during the pandemic. This is demonstrated by the lack of change in the number of patients presenting with both family history and positive screening. There were 44 patients before COVID, and 36 during, representing 20.1% and 25.2% respectively with no statistical difference between the temporal groups (*P* 0.16) despite the drop in colonoscopies (Table 3). It was also shown that within our study population, females (85.0%) were more likely to present with symptoms rather than screening, when compared to men (74.4%, *P* 0.01) (Table 4). However, amongst patients who were diagnosed using screening, there was no preference for screening type between genders, with 77.1% of males and 71.4% women choosing FIT testing (Table 4).

**Table 3.** Colorectal Cancer Family History Comparison Among Patients Diagnosed in Different Presentations Across Time Groups

	Positive family history	Negative family history
Total patients Before COVID	44 (20.1%)	175 (79.9%)
Total patients During COVID	36 (25.2%)	107 (74.8%)
*P* value	0.16	
Participants combined across time periods		
Symptomatic presentations	64 (81%)	218 (77.9%)
Screening presentations	15 (18.9%)	62 (22.1%)
*P* value	0.55	
FIT Screening	8 (57.1%)	43 (78.2%)
Procedural Screening	6 (42.9%)	12 (21.8%)
*P* value	0.08	

Family history status comparison across time periods in both number and method of presentations, as well as demographic data. For these comparisons, 10-14 participants omitted for lack of data. There was no significant difference between number of family history positive patients when compared across time periods (*P* 0.16). All *P* Values calculated using a Pearson Chi-Square Test.

**Table 4.** Method of Colorectal Cancer Patient Diagnosis Compared Across Sex Groups

	FIT screening	Procedural screening	Unknown screen type	Symptomatic presentation
Males	37 (17.5%)	11 (5.2%)	6 (2.8%)	157 (74.4%)
Females	15 (9.4%)	6 (3.8%)	3 (1.9%)	136 (85%)
*P* value	0.62			0.01

Demonstrating rates of symptomatic presentation, as well as screening types when available across sex groups. *P* Values calculated using a Pearson Chi-Square Test.

There were some challenges to data collection because of retrospective nature of the study. Compared with other studies, the total number of participants was lower, due to the smaller size of the regional cancer centre being studied. One area of potential bias is the sampling bias of our study, in that we collected data from the regional cancer centre to which patients are referred for systemic therapy. Patients who have their polyps or localised tumors fully resected during a colonoscopy would not be referred to the cancer centre, and therefore not be a subject within the study. Of note, the *n* number of the positive family history cohort was too low to make a temporal comparison, so therefore while it is presumed that the FIT tests in this population were performed during COVID, when colonoscopies were not available, there is a lack of certainty. This would result in skewing of the numbers to reduce the proportion of procedural screening, which may indicate that without the COVID pandemic the proportion of procedural screening in this group may be much higher. However, due to the low participant number in this particular subgroup it is difficult to draw firm conclusions about the rates of procedural screening in a temporal sub-analysis. Lastly, there may have been some patients who would have been screened in the end of 2019 but may have been classified under the COVID era cohort due to delay in diagnosis. This was mitigated by using the first date of diagnosis, rather than relying only on pathology results which are often delayed. Also, since this study primarily compared symptomatic to asymptomatic, this limits the impact of the above concern. In order to mitigate delayed diagnoses from impacting presentation rates, the study was extended from its initially planned 1 year timespan pre- and post-COVID, to an extended 18 months.

## Conclusions

Similar to other studies, a high proportion of patients referred to the Windsor Regional Cancer Centre before and during the COVID-19 pandemic were diagnosed symptomatically and not through screening programs.^17^ This indicates a need to further promote and improve participation in current screening programs. Further work needs to be done in promoting colorectal cancer screening, and encouraging self-referral screening programs or government-based blanket reminders for those who do not have family physicians. The demographic analysis of our study demonstrates a need to particularly target female patients, who are disproportionally presenting with symptoms, as well as ensuring that high risk groups such as those with family history are receiving appropriate, evidence-based screening to prevent colorectal cancer. Due to the maintenance of the proportion of patients diagnosed with screening during the pandemic, despite low colonoscopy participation, our data supports the effectiveness of FIT testing in detecting colorectal cancer, which may prove to be invaluable in expanding participation in colorectal screening programs due to its less-invasive and cost-effective approach. Future studies could be directed at providing further insight into the above findings, as well as extending the timeframe of study to better encompass delayed diagnoses missed during the pandemic. The findings in this study demonstrate consistency with new guidelines and mandates by Cancer Care Ontario, including the benefits of sending reminders directly to patients in order to improve participation in screening.^1^

## Data Availability

All raw data are available to share upon request. Please contact corresponding author during review process if desired.

## Appendix

### Abbreviations

CrC: Colorectal Cancer
EMR: Electronic Medical Record
CT: Computed Tomography
FIT: Fecal Immunochemical Testing
